# Role and Recent Advancements of Ionic Liquids in Drug Delivery Systems

**DOI:** 10.3390/pharmaceutics15020702

**Published:** 2023-02-20

**Authors:** Monu Kumar Shukla, Harshita Tiwari, Rachna Verma, Wen-Liang Dong, Shavkatjon Azizov, Brajesh Kumar, Sadanand Pandey, Deepak Kumar

**Affiliations:** 1 Department of Pharmaceutical Chemistry, School of Pharmaceutical Sciences, Shoolini University, Solan 173229, India; 2 Discovery Informatics Lab, Natural Products & Medicinal Chemistry Division, CSIR-Indian Institute of Integrative Medicine, Jammu 180001, India; 3 Department of Basic Sciences, Shoolini University, Solan 173229, India; 4 School of Pharmacy, Shandong University of Traditional Chinese Medicine, Jinan 250355, China; 5 Laboratory of Biological Active Macromolecular Systems, Institute of Bioorganic Chemistry, Academy of Sciences Uzbekistan, Tashkent 100125, Uzbekistan; 6 Department of Pharmaceutical Chemistry, Tashkent Pharmaceutical Institute, Tashkent 100015, Uzbekistan; 7 Department of Chemistry, TATA College, Kolhan University, Chaibasa 833202, India; 8 Department of Chemistry, College of Natural Sciences, Yeungnam University, Gyeongsan 38541, Republic of Korea

**Keywords:** drug delivery systems, ionic liquids, water solubility, biological macromolecules, biomedical applications

## Abstract

Advancements in the fields of ionic liquids (ILs) broaden its applications not only in traditional use but also in different pharmaceutical and biomedical fields. Ionic liquids “Solutions for Your Success” have received a lot of interest from scientists due to a myriad of applications in the pharmaceutical industry for drug delivery systems as well as targeting different diseases. Solubility is a critical physicochemical property that determines the drug’s fate at the target site. Many promising drug candidates fail in various phases of drug research due to poor solubility. In this context, ionic liquids are regarded as effective drug delivery systems for poorly soluble medicines. ILs are also able to combine different anions/cations with other cations/anions to produce salts that satisfy the concept behind the ILs. The important characteristics of ionic liquids are the modularity of their physicochemical properties depending on the application. The review highlights the recent advancement and further applications of ionic liquids to deliver drugs in the pharmaceutical and biomedical fields.

## 1. Introduction

Ionic liquids (ILs) are, generally, salts containing poorly coordinated anions and cations at room temperature (RT) or below 100 °C. ILs are also known as ionic fluids, ionic melts, liquid electrolytes, fused salts, liquid salts, ionic glasses, designer solvents, green solvents, and solvents of the future [1,2]. Since 1914, when Paul Walden published the first article on ionic liquids (ILs) concerning ethylammonium nitrate, ILs have gained prominence due to their unique physiochemical characteristics [3]. Wilkes and Zaworotkoin 1992, discovered an imidazolium-based IL that is both air- and water-stable at ambient temperature, which expanded the field’s applicability beyond synthetic chemistry and its application in biomedical fields. ILs are green solvents employed in green technology, and other methods can also be used for green synthesis [4,5,6,7,8,9]. Early research endeavored to develop ILs as eco-friendly, nonvolatile, stable solvents and noncombustible solvents. Ionic liquids (ILs) belong to a class of materials that are composed of ions and have distinctive properties such as high thermal stability, high solvating power, and low vapor pressure. These features of ILs are very useful in a variety of applications, including pharmaceutical drug discovery. One of the main applications of ionic liquids in drug discovery is the development of new solvents for chemical synthesis. ILs can be used as solvents for various reactions, including organic synthesis, and can also be used to enhance the solubility of compounds in aqueous media, which then produce it easier to purify and isolate compounds. This is also important in the early stages of drug discovery [10].

Another application of ILs in drug discovery is developing new drug delivery systems. ILs can be used to create nanoparticles and micelle structures to deliver drugs to specific target sites. Additionally, it can be used to create solid dispersions, which can improve the bioavailability and solubility of the drugs [10].

Latest studies have widened the field and characterized ILs as salts having lower melting points at approximately 100 °C and an infinite range of optimizable properties, such as volatility, toxicity, instability, and flammability [11,12,13].

**Figure 1:**
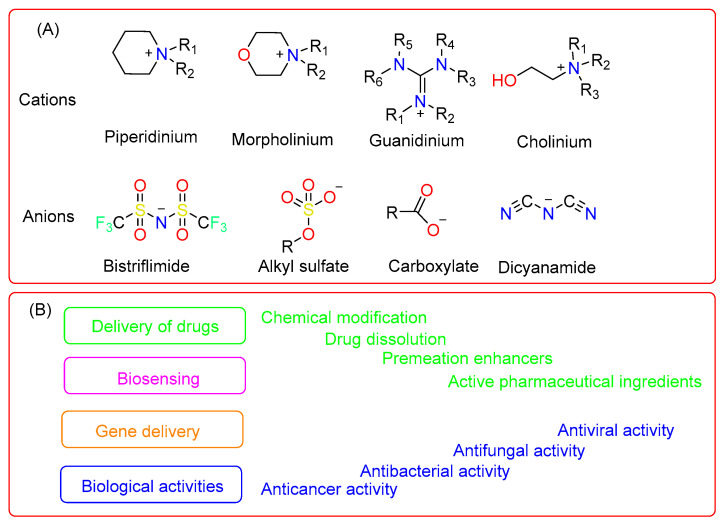
A list of different cationic and anionic molecules used in pharmaceuticals (**A**) and their various applications in different biomedical fields (**B**).

The ILs, as seen from the previously limited viewpoint as salts of imidazolium, pyrrolidinium, quaternary ammonium, phosphonium cations, or pyridinium, have expanded as additional cations, such as the bioinspired cations of guanidinium, cholinium and metal–based cations. They are combined with different anions to produce salts that satisfy the concept of ILs [14,15,16]. The emergence of more complex compounds supports the idea that the heterogeneity of the structure of an asymmetric sterical cation strongly interferes with both arranged packing inside a crystal lattice and the interaction with anions, which accounts for the compounds’ characteristically low melting point. Additionally, because of the expansion of the chemical space brought about by these recent developments, it is now possible to construct ILs with custom characteristics for both theoretical research and real-world applications. Application-driven studies of ILs are a thriving and expanding area of study with new horizons in therapeutics, particularly drug delivery (Figure 1A,B) [17].

The poorly soluble profiles of many drug candidates limit their bioavailability and lead to clinical failure; hence, it is essential to develop innovative solubilization and formulation methods to address this issue. One of the robust approaches is the application of ILs as a drug delivery method [18,19,20]. Moreover, coupling a pharmaceutically active cation with IL-based compounds resulted in improved solubility that permits effective absorption via several barriers to reach target cells [21,22]. This combination is also known as active pharmaceutical ingredient ionic liquids (API-ILs). 

However, empirical laboratory-scale findings demonstrate the IL strategy’s promise as a viable technique to address many issues, including the polymorphism linked to certain solid-state compounds and solubility [23,24]. This article will describe a summary of the different applications of ILs in the field of pharmaceutical sciences, thereby assisting researchers in developing a deeper understanding of ILs.

## 2. Ionic Liquids (ILs) Used as Solvents

Organic unsafe solvents should be substituted with more environmentally friendly ones that are less volatile and flammable. ILs are particularly helpful in this condition since they often have very little vapor pressure [25,26]. Additionally, the careful selection of cations and anions may result in unique interactions with specific solute groups, which are crucial for the solubility of compounds with some complexity such as active pharmaceutical ingredients and their precursors [27].

### 2.1. Pharmaceutical Drug Synthesis Using Ionic Liquids as an Alternative Medium

Organic solvents are often used in the industrial synthesis of medicinal compounds, which causes organic contamination of the finished product and frequently contains leftover contaminants [28]. For many organic modifications, including the production of therapeutic compounds, ILs have been investigated as a sustainable and alternative reaction medium (Table 1) [29]. Compared to the reactions found in traditional organic solvents, those in ILs are often quicker and simpler to conduct, and they typically do not need any sophisticated equipment or methods [29]. Cations such as (C4MIM) paired with the (NTf2), (BF4), or (PF6) anions have been employed as media in the synthesis of APIs, regardless of the wide varieties of alternative cations and anions that are now accessible in the IL toolbox [9]. The synthesis of nucleoside-containing antiviral drugs such as stavudine, trifluridine, and brivudine using ILs as reaction media is well documented in the literature. For instance, the synthesis of trifluridine in IL media resulted in a higher yield of approximately 90–91% with less reaction time between 20–25 min [29].

The feasibility to recover and reuse of solvents was proven in some of the earliest cases. Zhang et al. used 1-butyl-3-methylimidazolium hexafluorophosphate ((C4MIM)(PF6)) in order to synthesize hybrids of pyrimidine nucleoside; thiazolini-4-one [30]. These hybrids have the potential to be used as antiparasitic therapeutics. Zunita et al. utilized (Emim) Cl as a reaction medium, to establish a green efficacious synthesis of 5-hydroxymethylfurfural (HMF) from monosaccharides. In this case, the solvent can be reproduced multiple times without losing efficacy [31].

In addition, ILs have been used in the manufacture of compounds that show remarkable promise in the treatment of cancer. ILs (C4MIM)(X) (where X = BF_4_ or PF_6_) were utilized by Wolan et al. [32] during the preparation of L-4- boronophenylalanine (L-BPA) (a clinically recognized substance) for the treatment of boron-neutron capture. The cross-coupling reaction after just 20 min was carried out with pinacol borane employing protected p-iodophenylalanine with (C4MIM)(BF4), this made it possible to synthesize L-BPA and its analogs with excellent yields ranging from 82–89%. Using a unique and effective biocatalytic approach, Kurata et al. were able to produce numerous analogs of caffeic acid phenethyl ester (CAPE) which showed antiproliferative activity. The researchers got a conversion yield of 92% by using *Candida antarctica* lipase B (Novozyme-435) in 1-butyl-3-methylimidazolium bis(trifluoromethylsulfonyl)imide as the medium. The same result has been found as CAPE was produced with isooctane [33].

Traditional nonsteroidal anti-inflammatory drugs (NSAIDs) have also been synthesized using ILs as an alternate medium. The pravadoline was synthesized in this scenario and was carried out with imidazolium-based ILs using a combination of Friedel–Crafts reaction and the nucleophilic displacement process [34]. With 1-butyl-2,3-dimethylimidazolium hexafluorophosphate, the alkylation of 2-methylindole with 1-(N-morpholino)-2-chloroethane was accomplished (99% yield). In the reactions that are carried out in ILs as a reaction medium, neither catalysts nor conditions that are strictly anhydrous are needed [35]. Some other NSAIDs (such as (S)-naproxen) were produced by employing precursors, Ru-BINAP catalyst and (C4MIM)(BF4), immobilized in IL; the resulting optical yields were comparable to those homogeneous reactions [36]. Monteiro et al. established widely viable lipases and two native lipases from *Aspergillus terrus* and *Aspergillus niger,* which were then tested to check the kinetic resolution of (R,S)-ibuprofen throughout the systems that contain (C4MIM)(PF6) and (C4MIM)(BF4) [36].

**Table 1:** Application of ILs as a useful solvent for the production of drugs/molecules.

S. No.	Cations/Anions	Applications	Ref.
1.	Cations such as (C4MIM)in combination of anions (NTf2), (BF4), or (PF6)	Synthesis of APIs	[9]
2.	1-butyl-3-methylimidazolium hexafluorophosphate ((C4MIM)(PF6))	Synthesize hybrids of pyrimidine nucleoside; thiazolini-4-one	[30]
3.	(Emim)Cl	Synthesis of 5-hydroxymethylfurfural (HMF) from monosaccharides	[31]
4.	(C4MIM)(X) (where X = BF_4_ or PF_6_)	Preparation of L-4-boronophenylalanine (L-BPA)	[32]
5.	Pinacol borane protected p-iodophenylalanine with (C4MIM)(BF4)	Synthesis of L-BPA	[33]
6.	Imidazolium-based ILs in combination of Friedel-Crafts reaction and nucleophilic displacement process	Synthesis of Pravadoline	[34]
7.	Ru-BINAP catalyst and (C4MIM)(BF4)	Synthesis of (S)-naproxen	[36]
8.	(C4MIM)(PF6) and (C4MIM)(BF4)	Synthesis of (R,S)-ibuprofen	[36]

The use of ILs as substitute solvents for the production of various medicinal drugs is undoubtedly favorable. The applicability of ILs in place of organic hazardous solvents may often improve reaction conditions, speed up certain more difficult processes, and make it easier to purify and isolate the required product. From the perspective of the pharmaceutical industry, ILs may be a great solvent option for the production of certain particular drugs. The potential for IL recycling further expands the cost window for industrial-scale production of APIs in an IL environment [37].

### 2.2. Drug Delivery Using Ionic Liquids

It is possible to fine-tune the physicochemical characterization of ILs, which allows for efficient solutions to many issues associated with drug administration, such as limited drug insolubility and bioavailability. In this part, we will discuss how ILs are now being used, specifically as solubility promoters, permeation enhancers, and API-ILs [38].

#### 2.2.1. Enhancement of Permeability of Cell Membrane

**Figure 2:**
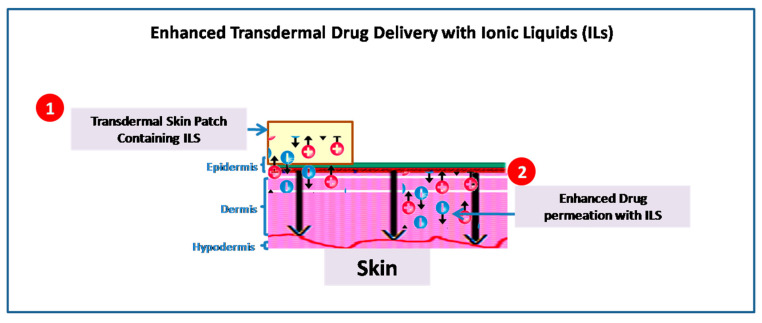
Illustration of permeation techniques of ILs applicable in transdermal drug delivery systems.

The drug delivery systems are important factors in determining a drug’s effectiveness and must be developed in a way that prevents the drug from being subjected to unfavorable metabolism and transports it to the target at a speed that is acceptable [39]. However, there are several physiological barriers that prevent the drugs from being transported effectively. For instance, the stratum corneum (SC) impermeability, which presents a barrier to chemical absorption, continues to provide a challenge to topical and transdermal drug delivery, providing alternatives to injectable and oral methods (Table 2) (Figure 2) [40,41]. Therefore, a variety of methods such as chemical enhancers, are used to overcome the obstacles and assure effective drug delivery [42]. In this context, ILs have garnered interest and are now being researched more and more as chemical enhancers to promote trans- and para-cellular drug transport [43]. 

Indeed, the experimental evidence and computer models both support the viability of adopting ILs to improve drug delivery. An empirical force field and molecular dynamics, for instance, revealed that the amphiphilic nature containing 1-octyl-3-methylimidazolium-based IL with a cationic head has been used together in the model cell membrane, which causes compromised structural integrity and increases the permeability of ammonia through the membrane like small polar molecules [44,45,46]. The experimental evidence supported the hypothesis that the hydrophilicity of imidazolium-based ILs is responsible for the cell membrane solvation to open channels to facilitate molecular passage [46]. 

Although these discoveries are connected with cytotoxicity and the IL is responsible for enhancing the permeation power of the biological membranes, it is conceivable to anticipate that these processes should allow for the delivery of drugs to their intended targets. ILs are responsible for detaching lipids from physiological structures like the SC. Such lipid-targeted actions do produce flaws that improve drug permeability. Diversified IL-based enhancers work differently using various mechanistic ways to settle the functional and structural integrity of physiological barriers, which facilitate effective drug movements, provided that their physicochemical qualities let them do so. Hydrophilicity, for example, facilitates paracellular transport by opening the tight junction, while hydrophobicity favors entering the epithelial membrane via partitioning to promote transcellular transport [47,48]. The polar enhancers develop interaction and are then inserted inside the lipid and protein regions of the SC, starting to cause fluidization depending on concentration, whereas the non-polar enhancers primarily target the lipid to break down the barriers and develop the channels for molecules to carry out the diffusion process [49].

As an alternative to aromatic imidazolium and pyridinium cations, considerable research is being conducted on alicyclic cations to uncover novel features. Monti et al. investigated 1,4-diazabicyclo[2.2.2]octane-, alicyclic pyrrolidinium-, and morpholinium-based ILs as promoters of transdermal layers to administer the hypertension-treating agent diltiazem known as nondihydropyridine calcium channel blocker [19]. The findings revealed that there are different drugs and that the ILs govern the transdermal permeation rate. It is also reported that the mono-cationic nature containing 1,4-diazabicyclo[2.2.2]octane beats the ineffective congener dicationic via more than twofold during delivery of diltiazem hydrochloride salts [19]. In addition, morpholinium-containing IL promotes the penetration of diltiazem HCl salts into the skin faster as compared to pyrrolidinium-based IL. The pyrrolidinium- and dicationic 1,4-diazabicyclo[2.2.2]octane-based ILs serve as enhancers, while diltiazem is supplied as a free base [19].

The indigenous toxicity of IL constructions motivates the investigation of the biomolecules as lead design towards the ILs’ next generation. The Cholinium cation, which is part of the cell membrane as a constituent of sphingomyelin and phosphatidylcholine and has been shown to readily permeate the cell membrane, is a developing bioinspired lead chemical. The coupling of cholinium cation with geranic acid (two equivalents) works as a pheromone in certain insects and also as a tyrosinase inhibitor in lemongrass [50]. Choline geranate (CAGE), which is a type 3 deep eutectic solvent (DES) for the delivery of drugs, is also an application of ILs [51]. DESs and ILs have low vapor pressure and high viscosity, but DESs are distinguished by their simple synthesis and a better understanding of the precursors’ toxicity. This study therefore examines CAGE and other DESs within ILs for the sake of simplicity. CAGE is a technique that serves as a chemical enhancer for transdermal distribution [16]. The capabilities of CAGE to deliver drugs are superior to those of controls, with the ability to boost the transport of mannitol and cefadroxil by 5 and 16-fold, respectively [52]. Other ILs such as tetraalkylphosphonium oleate, tetraalkylphosphonium hexanoate, choline oleate, and tetraalkylphosphoniumgeranate were developed as effective permeation enhancers, allowing a fivefold increase in the transport of cefadroxil through the dermis [52].

The property of molecules relies on the chemistry and structure of the IL, among other parameters. Indeed, the permeation of chemicals such as Transcutol^®^ and ethanol are some examples, but in these instances, choline hexanoate and choline urea are the two typical examples of DESs, which inhibit the transdermal permeation of mannitol [52,53]. The CAGE is reported for traditional promoters for fluorescein isothiocyanate-marked insulin, OVA, and BSA more effectively than the typical enhancers. The CAGE is also reported for better insulin transdermal delivery in male Wister rats after the topical administration of 10 U/mL of insulin-CAGE combination [54].

**Table 2:** Amphiphilic nature of various ILs for the enhancement of solubility and drug delivery.

S. No.	Amphiphilic Nature	Applications	Ref.
1.	1,4-diazabicyclo[2.2.2]octane-, alicyclic pyrrolidinium-, and morpholinium-based ILs	As promoters of transdermal layers to administer the hypertension-treating agent diltiazem	[19]
2.	Mono-cationic nature containing 1,4-diazabicyclo[2.2.2]octane	Beats the ineffective congener dicationic via more than two-folds during delivery of diltiazem hydrochloride salts	[19]
3.	Morpholinium-containing IL	Promotes the penetration of diltiazem HCl salts into the skin faster as compared to pyrrolidinium-based IL	[19]
4.	1-octyl-3-methylimidazolium-based IL	Improved drug delivery	[44,45,46]
5.	Imidazolium-based ILs	Cell membrane solvation to open channels to facilitate molecular passage	[46]
6.	Cholinium cation	Readily permeate the cell membrane, is a developing bioinspired lead chemical.	[50]
7.	Coupling of cholinium cation with geranic acid	Act as a pheromone in certain insects and also as a tyrosinase inhibitor in lemongrass	[50]
8.	Choline geranate (CAGE)	Used for the delivery of drugs	[51]
9.	Choline geranate (CAGE)	Ability to boost the transport of mannitol and cefadroxil by 5 and 16-fold, respectively	[52]
10.	ILs such as tetraalkylphosphonium oleate, tetraalkylphosphonium hexanoate, choline oleate, and tetraalkylphosphoniumgeranate	Developed as effective permeation enhancers, allowing a fivefold increase in the transport of cefadroxil through dermis	[52]
11.	Choline geranate (CAGE)	Promoters for fluorescein isothiocyanate-marked insulin, OVA, and BSA	[54]
12.	Choline geranate (CAGE)	Better insulin transdermal delivery in male Wister rats	[54]
13.	liquid salts generated from the carboxylic acid and aliphatic amines	Increase the drug’s penetration across physiological barriers	[55]

Similar to CAGE, additional liquid salts generated from the carboxylic acid and aliphatic amines increase the drug’s penetration across physiological barriers. In a recent publication on the mode of action of this family of ILs, the Kubota group investigated a variety of compounds developed by reacting isostearic or octanoic acid using di- or tri-isopropanolamine [55]. A combination of aliphatic amine and acid in equal proportion produces ILs, which exist alongside unreacted amine and carboxylic acid. The unreacted amine and acid correlated to the permeability enhancement function of the ILs, which are good permeation enhancers for hydrophilic drugs but function as retarders for hydrophobic drugs. Such activity differs from the well-known chemical accelerator Azone, which easily transfers tulobuterol from the skin but is inefficient for phenol red, suggesting that the ILs have a different mode of action. Based on this observation, Kubota et al. hypothesized that ILs produced from aliphatic amines and acids can develop self-assembled nanoparticles, having polar head regions that created a hydrophilic core while the alkyl chain provides the hydrophobic corona [55]. The suggested model shows that the ILs have very distinctive boosting properties. Besides this, the hydrophilic core is responsible for wrapping the hydrophilic phenol red very efficiently as compared to the hydrophobic tulobuterol, whilst the hydrophobic corona acts as a permeant to transport the drug (which is encapsulated) across the hydrophobic nature-bearing SC. Although the production of nanoparticles has not been explicitly validated, the self-assembling properties of ILs in the form of nanostructures have been well-established [29].

#### 2.2.2. Support for Low Solubility Drug Dissolution 

ILs have a good solvating capacity, which increases the solubility of poorly soluble drugs, resulting in the enhancement of the absorption and the bioavailability of the drugs. The transdermal administration of IL-assisted acyclovir is one of the most important examples of solubility enhancement with ILs [38]. Shamshina and colleagues overcame this obstacle via the dissolution of acyclovir in hydrophilic ILs, which contains strong hydrogen bond acceptors such as dimethylphosphate and dimethylimidazolium to increase its solubility (>10%) [56].

As a result of the hydrophobic SC acting as an obstacle to the hydrophilic IL, Moniruzzaman and colleagues were unable to detect any drug diffusion occurring via the skin [57]. However, the diffusion was performed by developing an IL-in-oil (IL/oil)-based microemulsion with the IL phase that embodies acyclovir and the continuous oil phase, which overcomes the developed hydrophobic hindrance to convey the payload [57].

The promising IL-assisted drug solubilization and penetration are not limited to the transdermal and topical modes of drug delivery, since research on oral routes has also shown efficacy. Utilizing the solvating ability of ILs, Porter, and colleagues improved the oral administration of water-insoluble drugs such as itraconazole and danazol. Different counteranions and the length of the alkyl linkers of a 1-hexyl-3-hexyloxycarbonylpyridinium-based ILs also affected the solubility [58]. Solubilities of itraconazole and danazol also increased to 5- and 3.6- fold, respectively, when the hydrophobic anion bis(trifluoromethane)sulfonamide ((NTf2)-) had been replaced with the hydrophilic anion dicyanamide ((N(CN)2)-. With the appropriate choice of the anion, it is feasible to integrate with the hydrogen bonding capacities of (N(CN)2) and the hydrophobicity of (NTf2) on a single platform to generate a solubility pattern that enables the dissolving of weakly water-soluble therapeutics. Alkyl sulfate anions, for instance, are hydrogen-bonding and hydrophobic; as a result, they facilitate the disintegration of danazol to a degree that is competitive with (N(CN)2]-. Notably, 1-hexyl-3-hexyloxycarbonylpyridinium-based ILs provide minimal benefits to administering water-insoluble drugs like fenofibrate besides the conventional excipients due to their modest solubility [59]. When the ILs constructed as self-emulsifying drug delivery systems (SEDDS), except the (N(CN)2)-containing ILs, which preserve the dissolution of danazol in simulated gastrointestinal fluid but had no benefit over the lipid-containing formulations which assure the drug absorption in rats [60]. The SEDDS obtained from (NTf2)- or (C10SO4)- results in a lower plasma concentration of danazol, but the SEDDS derived from (C18SO4)- is equivalent to the lipid-based approach owing to the enhanced uptake of the encapsulated drug by lipids [60].

#### 2.2.3. Designing New Active Pharmaceutical Ingredients-Ionic Liquids

According to estimates, solubility-limited bioavailability leads to the failure of 40–70% of potential drug candidates. The pharmaceutical industry is thus currently investigating several ways to enhance drug solubility and the pharmacological properties [61].

The idea behind API-IL is a novel way to deal with the issues that many drug candidates inherently have to achieve more effectiveness. This idea was first developed by Rogers and colleagues, who logically combined discrete anionic and cationic derivatives of legally available pharmaceuticals to regulate a variety of pharmacological cocktail characteristics, including solubility, stability, drug release, and bioavailability [62]. For instance, the Rogers group synthesized lidocaine docusate, a cheaper compound with better pharmacological characteristics and the potential for novel bioactive compounds. Lidocaine is a frequently used anesthetic for postsurgical and neuropathic pain [63]. Particularly, the better and prolonged therapeutic impact in a mouse model shows that the antinociceptive impact of lidocaine docusate is reported as better than that of lidocaine. The API-IL strategy changes the cellular level of lidocaine’s action mechanism. In pheochromocytoma (PC12) cells, lidocaine docusate has a better impact, which is different from the neuritic proliferation of lidocaine. Ranitidine docusate, made from sodium docusate and ranitidine hydrochloride, was also created via the API-IL method. Liquid ranitidine docusate’s effective design removes the polymorphism that exists in ranitidine [64].

New possibilities in pharmaceutics are made possible by Rogers and colleagues’ groundbreaking work on molecular engineering licensed pharmaceutically active compounds into API-ILs to obtain better pharmacological signatures [63,64]. The rising repository of API-ILs is proof that the area is receiving more attention due to the ease with which it is possible to access and describe ILs. The idea of improving the properties of licensed pharmaceuticals has been explored successfully many times. The pairing of the appropriate complementary acyclovir ion with the appropriate complementary cation—cholinium, tetrabutyl ammonium, tetrabutyl phosphonium, or tetramethyl-hexadecyl ammonium—or anion—chloride or docusate—to increase acyclovir’s solubility in water by over two orders of magnitude is a classic example. The API-ILs generated from acyclovir anions showed better water solubility as compared to the original drug, even with a 200fold increase in solubility from the hydrophobic tetrabutylphosphonium counterion. Additionally, the combination of the acyclovir cations with chlorides or docusate anions results in API-ILs that have an improved aqueous solubility profile when compared to neutral acyclovir, but no advantage has been found when compared to the acyclovir sodium salt form, whose solubility is exceptional for the docusate anion and similar to the chloride anion. The cholinium containing API-IL showed 650 times better solubility than the parent drug in intestinal simulated gastric fluid [65]. Additionally, the Marrucho group improved the pharmacological and physicochemical properties of the anions by combining cholinium with anions that are pharmaceutically active [65]. Particularly, API-ILs containing cholinium and anions of niflumic acid, nalidixic acid, pyrazinoic acid, picolinic acid, or 4-aminosalicylic acid have greater solubility in water, intestinal fluid, and gastric fluid [65,66]. Generally, increased in solubility increases the membrane permeability, which would further increase the bioavailability and therapeutic effectiveness of the compounds, but it might also result in overdosage, which would be harmful to important cellular functions. Despite the greater solubility API-ILs, the cytotoxicity of parent drugs for HepG2 hepatocellular carcinoma and Caco-2 colon cancer cells is comparable to the API-ILs of nalidixic, cholinium, niflumic, and pyrazinoic acid [66,67,68].

For a compound to carry out its therapeutic effects, cell membrane penetration could be necessary. The cell membrane permeability is governed by both extrinsic and intrinsic drug-related characteristics, with the hydrophilic/hydrophobic balance serving as a significant intrinsic determinant. A proper pairing of such an ionic agent with a complimentary ion provides the opportunity to regulate the balance, according to a logical design approach. By connecting the L-anion ampicillin to ammonium, pyridinium, phosphonium, or imidazolium cations, the Marrucho group successfully altered the hydrophilic/hydrophobic balance of the drug [25]. The API-ILs had a strong affinity to bind with the cell membrane in comparison to the drug and their sodium salt with an increased octanol/water partition coefficient (a measure of lipophilicity of the drug). Indeed, lipophilicity affects how compounds are transported and processed in the body as well as how they partition into the lipid bilayer. By manipulating the cationic head polarity to regulate the hydrophilicity, Marrucho and colleagues were able to control the hydrophobic/hydrophilic equilibrium [25]. Surprisingly, the octanol –water partition coefficient was unaffected by the ampicillin-containing API-ILs’ enhanced hydrophilicity. Even the sodium salts of ampicillin, which shares the same profile of water solubility as the cholinium containing API-IL, are less lipophilic, demonstrating the superior strategy of API-IL over the conventional form of the salt approach of attempting to regulate the solubility in pharmaceutical companies [69]. The API-IL technique is undoubtedly a more flexible tactic that enables synchronized management of several factors that affect a drug’s effectiveness, including hydrophilic/hydrophobic balance, drug absorption into solubility, systemic circulation, and biocompatibility [69].

Drug bioavailability, absorption, and therapeutic effectiveness all depend on the solubility of the drugs. By converting poorly water-soluble drugs into a lipophilic nature containing API-ILs, weakly water-soluble pharmaceuticals can be more effectively absorbed, resulting in a better bioavailability profile. By combining with carboxylate, alkyl sulfate, or (NTf2) anion, Porter and colleagues used this technique to increase the bioavailability of poorly soluble compounds such as cinnarizine and itraconazole, etc. [16,60]. In rats, oral treatment of the same had plasma concentrations of cinnarizine and itraconazole that are 2 and 20 times higher, respectively, than those receiving control formulations [70]. This team recently employed the API-IL technique to combine a small interfering RNA (siRNA) with a cation named benzyldimethylalkylammonium to concurrently adjust the siRNA’s physicochemical characteristics, skin permeability, and biocompatibility [71]. The technique improved the siRNA’s hydrophobic/hydrophilic ratio, turning the neat siRNA hydrophilic into the lipophilic form of siRNA-based API-IL. It is anticipated that increased lipophilicity would improve skin permeability and siRNA absorption. Indeed, tests using confocal laser scanning microscopes and Franz diffusion cells unmistakably demonstrated the improvement of these features. As an example, after transformation to API-IL, siRNA uptake via human adult epidermal keratinocytes (HEKa) and the proportion of siRNA transported via the epidermis of pig skin, both were improved [71]. The percent delivery has enhanced by switching the octyl position on the cationic element of benzyldimethylalkylammonium in the API-IL to tetradecyl. Additionally, the kind of the alkyl group affects biocompatibility since switching the octyl group for a stearyl pr tetradecyl group makes HEKa more hazardous. It is also reported that the neat siRNA has more biocompatibility with HEKa than with API-ILs, which supports the hypothesis that hydrophobicity and cytotoxicity are positively correlated. However, the API-IL platforms address skin conditions better than the plain RNA, which is a benefit [71,72].

### 2.3. Remedies for Pharmaceutical Drug Polymorphism-Related Problems

An appropriate and well-described method to combat the phenomena of polymorphism, and crystallization tendencies of APIs is the synthesis of salts of specific active chemicals. However, amorphous forms, co-crystallinity, and polymer-embedded drugs have all been investigated as solutions to or workarounds for conventional issues, like the spontaneous polymorphic transition of drug forms carrying a crystalline nature. These methods may alter an effective dosage form into a deadly dose by altering the solubility of the active component, which is a serious issue for drug designers [73].

Polymorphism may occur in pure compounds, salts, and therapeutic drug candidates. There are ways to forecast this phenomenon for each chemical, despite current attempts to better comprehend crystal polymorphism in medicinal drugs. Some examples of crystallized forms of pharmaceutical compounds having polymorphism issues such as indinavir sulphate ethanolate, itraconazole succinic acid cocrystal, and sertraline hydrochloride. The crystal polymorphism and salvation state have a direct impact on a medicinal product’s price [73,74]. Expensive product failures and drawn-out patent disputes serve as examples of this circumstance. The failure of the ritonavir capsule product in 1998, which was justified with the help of resolving the crystal structures of the drug, is one of the most well-known instances [74]. A novel polymorphism structure of the well-known anticancer drug 5-fluorouracil was characterized as an unexpected metastable form [75]. For the distribution of APIs, pharmaceutical firms generally use solid, mostly crystalline forms because of their purity, thermal stability, simplicity of manufacture, and handling [76].

While significantly less prevalent, liquid drug formulations are often based on eutectic mixtures. A few of the polymorphism issues related to solids may be avoided by using an active compound in liquid form. Other comparable strategies have been devised using liquid formulations as eutectic mixtures, but they may dilute the APIs due to the presence of significant amounts of inert filler in the formulation. In a similar vein, the potential for solubilizing the APIs as biocompatible ILs may open up new possibilities for drug delivery and therapeutic strategies. The usage of liquid salts, ideally having melting temperatures below room temperature, is pertinent from the perspective of the pharmaceutical business [77]. Some synthetic techniques, like selecting cations with a low inclination to crystallize or ions with sufficient diffusive charge, may be created to lower the melting point of salts. For instance, 1-ethyl-3-methylimidazolium chloride is an organic salt which has a melting point at approximately 77–79 °C, which may be reduced up to 21 °C by simply substituting a dicyanamide anion for the chloride [78]. In conclusion, it has been stated that the development of API-ILs or the straightforward dissolving of APIs into the biocompatible ILs is a unique and effective method for eliminating polymorphism and therefore increasing drug effectiveness.

## 3. Ionic Liquids with Metals Added to Detect Biomolecules

When a metal core is incorporated into an organic molecule, the intrinsic stability and processability of organic molecules are combined with the distinctive magnetic, optical, and redox properties of metals. To provide liquid salts additional roles, metal-containing ILs are developing as alternatives to ILs that are only organic [79]. Due to difficulties in synthesizing organometallic and coordination compounds, progress in this growing subject is gradual, but it is interesting and promising since it is possible to create molecules with unusual features like magnetism and luminescence. There are a few studies that outlined the production of metal-based ILs, but information on their uses is limited [80]. However, Benjamin et al. developed a salophen-type IL for sensing glucose nonenzymatically that contains cobalt (Co) [81]. An essential component of managing diabetes mellitus, which claimed the lives of 1.5 million individuals globally in 2012 due to life-threatening complications brought on by increased blood glucose levels, is glucose sensing. The metal-containing nonenzymatic technology is cost-effective, reliable, and simple to manufacture, which makes it an appealing alternative to enzyme-based glucose sensors when looking for novel glucose biosensors [81,82,83]. This new technique has the flexibility that the enzyme-based technology lacks since it can adjust the redox activity of a transition metal by choosing the right ligand. Therefore, adding metals to ILs should provide a synergy between the IL’s physicochemical characteristics, including ionic conductivity, and the metal’s redox activity in addition to synchronously controlling these characteristics.

Salophen IL (salophen IL is N,N-(o-phenylene)bis-[(3-ethyl-1H-benzimidazole-1-ium-1-yl)methylene hexafluoro-phosphato] salicylideneimine), upon an electrochemically decreased graphene oxide, results in accumulation on a screen printed carbon electrode. The modified electrode (mostly in the context of glucose) produces a greater anodic current, having an onset that is consistent with the Co^2+^/^3+^ redox pair rather than an unmodified electrode. The electrochemical oxidation of Co^2+^ to Co^3+^, which oxidizes the glucose into gluconolactone while being reduced to Co^2+^, is the mechanism by which glucose is sensed. If glucose is available, the ionically conductive IL allows for efficient electron transfer to power a redox cycle and modified electrode could be able to detect glucose in actual human urine and blood serum [81]. 

## 4. Ionic Liquid Toxicity

The idea of “ILs as green chemicals” originated from the fact that ILs are often nonvolatile and nonflammable, unlike organic solvents. In a chemical or biological process, the replacement of a volatile organic solvent with a nonvolatile IL reduces the risk of inhalation exposure, flammability, and environmental contamination [76].

Empirical evidence indicates that many ILs are hazardous to a variety of biological forms, including nucleic acids and multicellular creatures. Studies conducted in in silico, in vivo, and in vitro have elucidated the complexities and molecular basis of ILs’ toxicity, which improved our knowledge about the crucial factors that regulate toxicity. Due to the structural variety of ILs, the influence of the different cationic head groups is convoluted and a clear trend is very difficult to explain. The observable difference in toxicity of the cationic head groups tetrabutylphosphonium (Log IC_50_ 2.58–2.64 l M) and tetrabutylammonium (Log IC_50_ 2.25–2.35 l M) to inhibit the activity of acetylcholinesterase of electric eel; however, the charge density of cations influences the toxicity degree [84]. The relatively higher atomic radius of phosphorus in contrast to nitrogen lowers the charge density of cations of the phosphonium head group, due to decreasing the electrostatic interactions with endogenous anionic biomolecules. These interactions are important for the toxicity of cationic bioactive compounds like ILs. Therefore, theoretically, altering the charge density by cationic head group replacements may provide a method for adjusting the toxic effects profiles of ILs [84].

The impact of the group carrying dimethylamino on the toxic effects of pyridinium-containing ILs shows how the cationic head group substituents may be tuned for toxicity. The enzyme acetylcholinesterase and the promyelocyticleukemia rat cell line IPC-81120 are more sensitive to 4-dimethylaminopyridinium-based IL than to pyridinium. It is plausible that an electron-donor group (such as dimethylamino) at the para position could alter the solubility or electrical properties of the molecules to render them nontoxic [85].

According to the substituent effect, 4-dimethylaminopyridinium is a greater lipophilic agent than pyridinium. The bioactivity of the substituent may also be changed by interactions with endogenous compounds. Due to this, the alicyclic morpholinium cation has a lower inhibitory potential on acetylcholinesterase than aromatic cations such as pyridinium and imidazolium, in addition to the alicyclic cations piperidinium and pyrrolidinium. The ethereal oxygen can form hydrogen bonds with endogenous water molecules in the morpholinium cation, which is somewhat less lipophilic, and causes less engagement with endogenous hydrophobic materials [85]. This is opposed to less active cationic head groups like pyridinium and imidazolium, where the side chain greatly influences the toxicity with the more bioactive cationic head group, including quinolinium or 4-dimethylaminopyridinium, which mostly determines the toxicity. On the promyeloticleukaemia rat cell line IPC-81, for instance, the toxic effects of pyridinium, imidazolium, pyrollidinium, piperidinium, and morpholinium are significantly comparable, indicating a lesser effect from the head group containing cations. Additionally, the amount of toxicity is greatly influenced by the branching of the compound having the cationic head, although this impact differs between nonaromatic and aromatic head groups [85]. For instance, Kurnia et al. discovered that branching of the compound improves the toxic effects of nonaromatic piperidinium and pyrrolidinium-based ILs while decreasing the toxic effects of aromatic pyridinium and imidazolium-containing ILs toward *V. fisheri* [86]. The EC_50_ values of the aromatic imidazolium and pyridinium-containing ILs rise in water solubility, as a result, which corresponded with the heuristic rule that hydrophobicity promotes toxicity, and thus adds to their toxicity against *V. fisheri*. The nonaromatic piperidinium and pyrrolidinium-derived ILs, however, violate the norm by showing no correlation between EC_50_ and solubility, indicating that mechanisms other than hydrophobicity control the toxicity of this series [86].

The investigation of biomaterials such as cholinium as cationic head groups is motivated by the demand for benign ILs. Some bioinspired cholinium-based ILs, such as pyridinium and imidazolium-based ILs, have less harmful effects on a variety of living forms than traditional ILs. The least amount of a cholinium-based IL which suppresses the activity of acetylcholinesterase and the visible growth of *E. coli*, *L. monocytogenes*, *S. enteritidis,* and *S. aureus,* is an order of magnitude greater than that of an imidazolium-based IL [87]. Thus, throughout time, various imidazolium-based ILs have been reported of IL-functionalized quantum dots (QDs) for fluorescent and ion sensing inks in bioimaging and biosensing applications, respectively [88]. The applicability of the QDs with fluorescent properties and ILs’ protein affinity have been utilized to synthesize silica-capped imidazolium-based IL-CdTe QDs, which possess selective fluorescent detection and recognition of hemoproteins via covalent interactions of ILs-protein in biological fluids [89].

## 5. Ionic Liquid-Based New Active Pharmaceutical Ingredients

### 5.1. Pharmaceutical Salts Ionic Liquid (API-ILs) Synthesis

Generally, the synthetic procedure is a straightforward method to change the properties of the compounds using ionizable functional groups to eliminate undesired traits of the original drug. Prospective ions with poor symmetry and diffuse charge, which also describes several common APIs, are used in the choice of ion pairs to create ILs. Even the heterocycles with nitrogen that are now employed in ILs are typically found in APIs or their precursors. Nayl et al. reported the use of pyridinium-based dihydroxy ionic liquid ((Py-2OH) OAc) to improve the synthesis process and yield more potent anticancer compounds [90].

**Figure 3:**
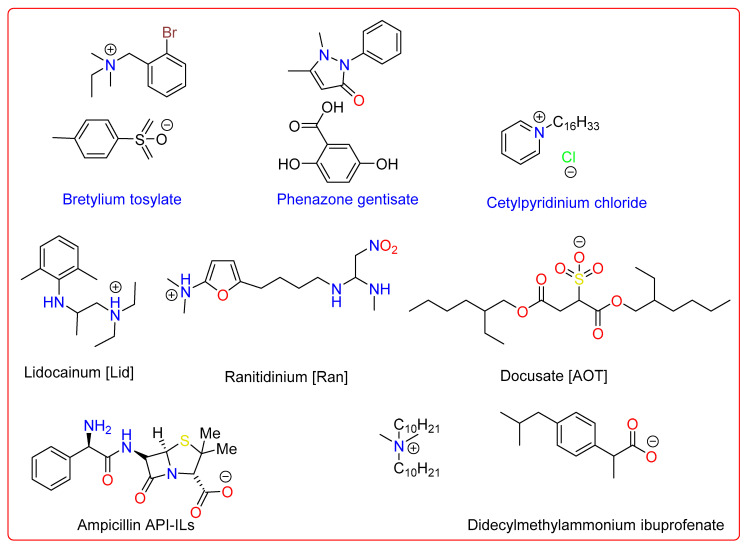
Examples of some active pharmaceutical ingredients-ionic liquids (API-ILs).

Numerous kinds of organic pharmaceutical salts have been established in the last century to alter the physicochemical or biological characteristics of compounds that was initially neutral. Figure 3 illustrates several organic pharmaceuticals that may be categorized as ILs. This group of pharmacological salt pairs—those that exhibit IL characteristics and comprise both anion and cation as active ingredients—has long been recognized in the literature. Examples of API-ILs include the antiseptic medicine cetylpyridinium chloride (melting point (m.p.) 77 °C, 1981), the anti-fibrillatory and antiarrhythmic drug bretylium (m.p. 86 °C, 1978), and the analgesic, anti-inflammatory, and antipyretic drug phenazone gentisate (m.p. 88 °C, 1951). Among examples of API-ILs, didecyldimethylammoniumibuprofenate, a local anesthetic and antiarrhythmic, lidocainium docusate, and ranitidine docusate, a histamine H2 receptor antagonist, were obtained using straightforward metathesis processes (Figure 3) [91].

The majority of API-IL syntheses that have been documented in the literature include metathesis reactions. Individually dissolved in a chosen solvent (such as water, ethanol, methanol, or acetone), the cation and anion are in their commercially available salt forms. The solution is then agitated at an ambient temperature or heated to between 50 and 100 °C (if required). Because inorganic salts (such as NaCl) are present, this synthesis technology has certain limits in terms of the API-ILs’ ultimate purity [92]. However, these constraints may be overcome by selecting the right solvent or by utilizing additional purifying techniques. An extraction procedure using apolar solvents, such as chloroform or dichloromethane, is employed in API-IL purification whenever the inorganic salt is only the least part soluble in organic solvents. After that, the organic phase is washed with water to remove the inorganic salt (for example, NaCl, which can be verified by a silver nitrate test), and the solvent is eliminated using a rotary evaporator. The finished product is put on a high vacuum line in the last stage to get rid of any remaining solvent. In a few instances, further purification is mentioned, mostly to get rid of more halides [92].

The presence of residual contaminants significantly affects the physical, chemical, thermal, and biological characteristics of API-ILs produced via metathesis processes. The creation of innovative API-ILs depends heavily on the hunt for sustainable and more effective synthesis methods. ILs improve the physicochemical property and optimize the use of these substances. However, additional research is required to completely comprehend their effects on biological and pharmacological properties before they can be used on an industrial scale [93,94].

### 5.2. Salts of Protic Pharmaceuticals

Many significant APIs are deprotonated or protonated to create the frequently used salts with the appropriate pKa variations, rather than being permanent ions. The classification of protic ILs is a topic of significant discussion. In reality, drugs with modest levels of ionization offer many benefits over those that are completely ionized, most notably a better capacity to pass membranes [95]. 1-Methylhexylammonium salicylate is an example of a pharmaceutically useful, partly ionized IL. A nasal decongestant 1-methylhexylamine with 10.5 pKa value, and salicylic acid with 2.98 pKa value, were combined to develop a liquid with ambient temperature having the glass transition at 40 °C (pKa = 7.52) [95].

**Figure 4:**
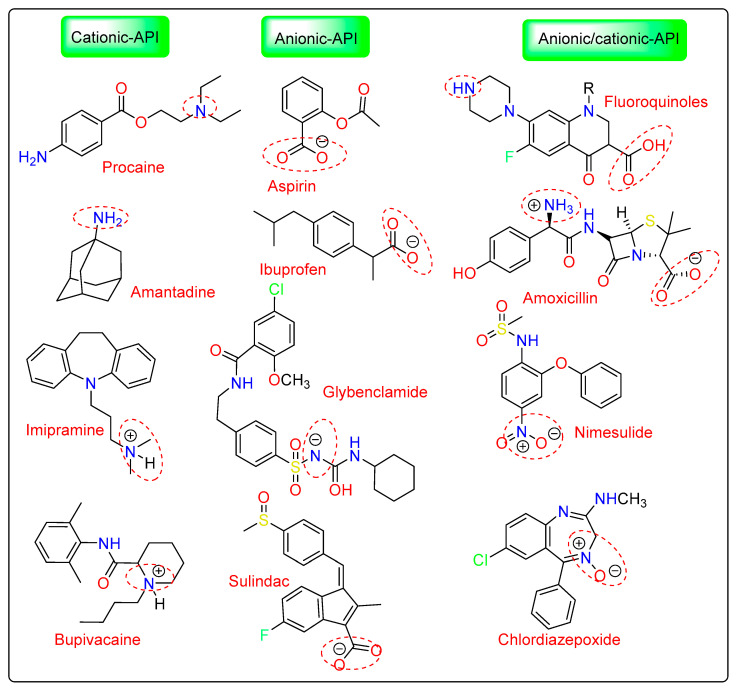
Some promising IL-assisted drugs with good solubilization and penetration properties reported for transdermal and topical modes of drug delivery.

Nasal decongestants are made from tuaminoheptane, a primary amine base that has 10.50 pKa value. Amantadine is also a primary amine base with a pKa value of 10.10 (Figure 4). Nine out of the thirteen synthesized compounds meet the criteria for ILs having below 100 °C of melting points, with five of these compounds being ILs at ambient temperature. The high melting points are seen in all salts that include the amantadine base. All ((EtOH)PYRH)+-containing ILs have melting points under 100 °C, however, there are no clear patterns in the melting points of (NTH3)+-based ILs. To fully grasp the significance of protic ILs in therapeutic formulations, it is important to examine their chemical and thermal stability for each scenario [23].

### 5.3. Assessing Enhanced Properties of API-IL

Converting the active pharmaceutical ingredient (API) to ionic liquid is a tool to modulate the physicochemical properties such as solubility and stability of API. It is well-studied that most of the APIs change from amorphous to crystalline form during storage, which greatly affects their physicochemical properties and efficacy [21,22,23].

In this context, API-IL can be used to address the issue of polymorphic conversion. One of the examples is the improvement of the solubility of water-insoluble NSAID ibuprofen by using the (C_2_OHmim)+ cation. A combination of ibuprofen with on (C_2_OHmim)+ cation resulted in the solubility of ibuprofen being increased by 105 times [96]. Another application is the delivery of a combination of NSAIDs with local anesthetics. API-ILs have been used to increase the lipophilicity of topical NSAIDs with local anesthetics. API-ILs are an indisputable effective tool to produce liquid versions of therapeutic molecules [97]. The proton-transfer action of protic ILs (which is made up of active pharmaceutic anions) was explored by Stoimenovski et al. in model membrane transport [95]. In several publications, the stability of the ionic formulation of acidic drugs was considered troublesome. Despite the rapid growth of this theme, there is still more work to be done, especially in terms of evaluating the unique qualities of API-ILs [97,98].

## 6. Conclusions and Recommendations

The goal of this study is to summarize and inspire ongoing ionic liquid (ILs) research that is motivated by biological applications. The characteristics of ILs may be modified to a variety of biological purposes with the aid of great structural diversity of the ions and their easy coupling properties. Advances in this area have shown how ILs may improve drug permeability across physiological barriers, encourage the dissolution of pharmaceuticals with poor solubility, and provide a method for designing API-ILs. The findings have been quite positive, with this IL-hallmarks strategy of improved bioavailability and effectiveness. Metal-containing ILs showed better applications in biosensing as well as in the detection of glucose. This may develop as a tool for the treatment and diagnosis of different diseases with the aid of transition metals and can enhance different properties including luminescence, redox activity, and magnetism. ILs can be viewed as a viable strategy for addressing a variety of issues, including the bioavailability of pharmaceuticals, anti-infective, and biosensors in the biomedical industry. Considering the magnetic, optical, and radioactive capabilities of metals, we believe the developing area of ILs incorporating metals may provide fresh prospects, such as in bioimaging. Toxicity is a big risk while utilizing ionic liquid. However, toxicity can be reduced through the rational design of formulation. In conclusion, ILs constitute an unexplored field with enormous therapeutic potential.

## Data Availability

Not applicable.

## References

[1] Lei Z., Chen B., Koo Y.M., MacFarlane D.R. (2017). Introduction: Ionic liquids. Chem. Rev..

[2] Procopio D., Siciliano C., Trombino S., Dumitrescu D.E., Suciu F., Di Gioia M.L. (2022). Green solvents for the formation of amide linkages. Org. Biomol. Chem..

[3] Walden P. (1914). Molecular weights and electrical conductivity of several fused salts. Bull. Acad. Imper. Sci..

[4] Wilkes J.S., Zaworotko M.J. (1992). Air and water stable 1-ethyl-3-methylimidazolium based ionic liquids. J. Chem. Soc. Chem. Commun..

[5] Rao R.V., Patil K.T., Kumar D., Gupta M.K., Shin D.-S. (2022). Mild and efficient one-pot synthesis of (E)-styrylperfluoroalkyl ketone from styrene. Results Chem..

[6] Kharissova O.V., Kharisov B.I., Oliva G.C.M., Méndez Y.P., López I. (2019). Greener synthesis of chemical compounds and materials. R. Soc. Open Sci..

[7] Liu G., Zhong R., Hu R., Zhang F. (2012). Applications of ionic liquids in biomedicine. Biophys. Rev. Lett..

[8] Rao V.R., Patil K.T., Kumar D., Sebastian S., Gupta M.K., Shin D.-S. (2022). Facile metal-free visible-light-mediated chlorotrifluoromethylation of terminal alkenes. Mon. Chem..

[9] Hayes R., Warr G.G., Atkin R. (2015). Structure and nanostructure in ionic liquids. Chem. Rev..

[10] Moshikur R.M., Goto M. (2021). Ionic Liquids as Active Pharmaceutical Ingredients (APIs). Application of Ionic Liquids in Drug Delivery.

[11] Shi W., Luebke D.R. (2013). Enhanced gas absorption in the ionic liquid 1-n-hexyl-3-methylimidazolium bis (trifluoromethylsulfonyl) amide ([hmim][Tf2N]) confined in silica slit pores: A molecular simulation study. Langmuir.

[12] Greaves T.L., Drummond C.J. (2008). Ionic liquids as amphiphile self-assembly media. Chem. Soc. Rev..

[13] Liu Y., Chen J., Li D. (2012). Application and Perspective of Ionic Liquids on Rare Earths Green Separation. Sep. Sci. Technol..

[14] Egorova K.S., Ananikov V.P. (2014). Toxicity of ionic liquids: Eco (cyto) activity as complicated, but unavoidable parameter for task-specific optimization. ChemSusChem.

[15] Dinis T.B.V., e Silva F.A., Sousa F., Freire M.G. (2021). Advances Brought by Hydrophilic Ionic Liquids in Fields Involving Pharmaceuticals. Materials.

[16] Agatemor C., Ibsen K.N., Tanner E.E.L., Mitragotri S. (2018). Ionic liquids for addressing unmet needs in healthcare. Bioeng. Transl. Med..

[17] Adawiyah N., Moniruzzaman M., Hawatulaila S., Goto M. (2016). Ionic liquids as a potential tool for drug delivery systems. MedChemComm.

[18] Zandu S.K., Chopra H., Singh I. (2020). Ionic Liquids for Therapeutic and Drug Delivery Applications. Curr. Drug Res. Rev..

[19] Monti D., Egiziano E., Burgalassi S., Chetoni P., Chiappe C., Sanzone A., Tampucci S. (2017). Ionic liquids as potential enhancers for transdermal drug delivery. Int. J. Pharm..

[20] Handa M., Almalki W.H., Shukla R., Afzal O., Altamimi A.S.A., Beg S., Rahman M. (2022). Active pharmaceutical ingredients (APIs) in ionic liquids: An effective approach for API physiochemical parameter optimization. Drug Discov. Today.

[21] Ford L., Tay E., Nguyen T.-H., Williams H.D., Benameur H., Scammells P.J., Porter C.J.H. (2020). API ionic liquids: Probing the effect of counterion structure on physical form and lipid solubility. RSC Adv..

[22] Aungst B.J. (2017). Optimizing Oral Bioavailability in Drug Discovery: An Overview of Design and Testing Strategies and Formulation Options. J. Pharm. Sci..

[23] Censi R., Di Martino P. (2015). Polymorph Impact on the Bioavailability and Stability of Poorly Soluble Drugs. Molecules.

[24] Berthod A., Ruiz-Angel M.J., Carda-Broch S. (2008). Ionic liquids in separation techniques. J. Chromatogr. A.

[25] Marrucho I.M., Branco L.C., Rebelo L.P.N. (2014). Ionic liquids in pharmaceutical applications. Annu. Rev. Chem. Biomol. Eng..

[26] Hansen B.B., Spittle S., Chen B., Poe D., Zhang Y., Klein J.M., Horton A., Adhikari L., Zelovich T., Doherty B.W. (2021). Deep Eutectic Solvents: A Review of Fundamentals and Applications. Chem. Rev..

[27] Grodowska K., Parczewski A. (2010). Organic solvents in the pharmaceutical industry. Acta Pol. Pharm..

[28] Pedro S.N., RFreire C.S., Silvestre A.J.D., Freire M.G. (2020). The Role of Ionic Liquids in the Pharmaceutical Field: An Overview of Relevant Applications. Int. J. Mol. Sci..

[29] Kumar V., Malhotra S.V. (2008). Synthesis of nucleoside-based antiviral drugs in ionic liquids. Bioorg. Med. Chem. Lett..

[30] Zhang X., Li X., Li D., Qu G., Wang J., Loiseau P., Fan X. (2009). Ionic liquid mediated and promoted eco-friendly preparation of thiazolidinone and pyrimidine nucleoside-thiazolidinone hybrids and their antiparasitic activities. Bioorg. Med. Chem. Lett..

[31] Zunita M., Yuan D.M., Syafi’ Laksono A. (2022). Glucose conversion into hydroxymethylfurfural via ionic liquid-based processes. Chem. Eng. J. Adv..

[32] Wolan A., Zaidlewicz M. (2003). Synthesis of arylboronates by the palladium catalysed cross-coupling reaction in ionic liquids. Org. Biomol. Chem..

[33] Kurata A., Kitamura Y., Irie S., Takemoto S., Akai Y., Hirota Y., Fujita T., Iwai K., Furusawa M., Kishimoto N. (2010). Enzymatic synthesis of caffeic acid phenethyl ester analogues in ionic liquid. J. Biotechnol..

[34] Earle M.J., Seddon K.R., McCormac P.B. (2000). The first high yield green route to a pharmaceutical in a room temperature ionic liquid. Green Chem..

[35] Earle M.J., Seddon K.R. (2000). Ionic liquids. Green solvents for the future. Pure Appl. Chem..

[36] Monteiro A.L., Zinn F.K., de Souza R.F., Dupont J. (1997). Asymmetric hydrogenation of 2-arylacrylic acids catalyzed by immobilized Ru-BINAP complex in 1-n-butyl-3-methylimidazolium tetrafluoroborate molten salt. Tetrahedron Asymmetry.

[37] Kudłak B., Owczarek K., Namieśnik J. (2015). Selected issues related to the toxicity of ionic liquids and deep eutectic solvents—A review. Environ. Sci. Pollut. Res..

[38] Shamshina J.L., Barber P.S., Rogers R.D. (2013). Ionic liquids in drug delivery. Expert Opin. Drug Deliv..

[39] Carvalho P.O., Cass Q., Calafatti S.A., Contesini F., Bizaco R. (2006). Review—Alternatives for the separation of drug enantiomers: Ibuprofen as a model compound. Braz. J. Chem. Eng..

[40] Sidat Z., Marimuthu T., Kumar P., du Toit L.C., Kondiah P.P.D., Choonara Y.E., Pillay V. (2019). Ionic Liquids as Potential and Synergistic Permeation Enhancers for Transdermal Drug Delivery. Pharmaceutics.

[41] Alqahtani M.S., Kazi M., Alsenaidy M.A., Ahmad M.Z. (2021). Advances in Oral Drug Delivery. Front. Pharmacol..

[42] Haque T., Talukder M.M.U. (2018). Chemical Enhancer: A Simplistic Way to Modulate Barrier Function of the Stratum Corneum. Adv. Pharm. Bull..

[43] Gupta R., Dwadasi B.S., Rai B., Mitragotri S. (2019). Effect of Chemical Permeation Enhancers on Skin Permeability: In silico screening using Molecular Dynamics simulations. Sci. Rep..

[44] Lu B., Liu T., Wang H., Wu C., Chen H., Liu Z., Zhang J. (2022). Ionic liquid transdermal delivery system: Progress, prospects, and challenges. J. Mol. Liq..

[45] Kumar S., Scheidt H.A., Kaur N., Kaur A., Kang T.S., Huster D., Mithu V.S. (2018). Amphiphilic Ionic Liquid-Induced Membrane Permeabilization: Binding Is Not Enough. J. Phys. Chem. B.

[46] Sindhu A., Bhakuni K., Sankaranarayanan K., Venkatesu P. (2020). Implications of Imidazolium-Based Ionic Liquids as Refolding Additives for Urea-Induced Denatured Serum Albumins. ACS Sustain. Chem. Eng..

[47] Hmingthansanga V., Singh N., Banerjee S., Manickam S., Velayutham R., Natesan S. (2022). Improved Topical Drug Delivery: Role of Permeation Enhancers and Advanced Approaches. Pharmaceutics.

[48] Gao L., Lu C., Ma S., Yan X., Jiang X., Wu X., He G. (2020). Flexibly crosslinked and post-morpholinium-functionalized poly (2, 6-dimethyl-1, 4-phenylene oxide) anion exchange membranes. Int. J. Hydrog. Energy.

[49] Laksitorini M., Prasasty V.D., Kiptoo P.K., Siahaan T.J. (2014). Pathways and progress in improving drug delivery through the intestinal mucosa and blood–brain barriers. Ther. Deliv..

[50] Yu A.S.L. (2017). Paracellular transport as a strategy for energy conservation by multicellular organisms?. Tissue Barriers.

[51] Boch R., Shearer D.A. (1964). Identification of Nerolic and Geranic Acids in the Nassanoff Pheromone of the Honey Bee. Nature.

[52] Smith E.L., Abbott A.P., Ryder K.S. (2014). Deep Eutectic Solvents (DESs) and Their Applications. Chem. Rev..

[53] Ibsen K.N., Ma H., Banerjee A., Tanner E.E.L., Nangia S., Mitragotri S. (2018). Mechanism of Antibacterial Activity of Choline-Based Ionic Liquids (CAGE). ACS Biomater. Sci. Eng..

[54] Tanner E.E.L., Ibsen K.N., Mitragotri S. (2018). Transdermal insulin delivery using choline-based ionic liquids (CAGE). J. Control. Release.

[55] Kubota K., Shibata A., Yamaguchi T. (2016). The molecular assembly of the ionic liquid/aliphatic carboxylic acid/aliphatic amine as effective and safety transdermal permeation enhancers. Eur. J. Pharm. Sci..

[56] Shamshina J.L., Cojocaru O.A., Kelley S.P., Bica K., Wallace S.P., Gurau G., Rogers R.D. (2017). Acyclovir as an ionic liquid cation or anion can improve aqueous solubility. ACS Omega.

[57] Moniruzzaman M., Tamura M., Tahara Y., Kamiya N., Goto M. (2010). Ionic liquid-in-oil microemulsion as a potential carrier of sparingly soluble drug: Characterization and cytotoxicity evaluation. Int. J. Pharm..

[58] Porter C.J., Pouton C.W., Cuine J.F., Charman W.N. (2008). Enhancing intestinal drug solubilisation using lipid-based delivery systems. Adv. Drug Deliv. Rev..

[59] Williams H.D., Sahbaz Y., Ford L., Nguyen T.-H., Scammells P.J., Porter C.J.H. (2014). Ionic liquids provide unique opportunities for oral drug delivery: Structure optimization and in vivo evidence of utility. Chem. Commun..

[60] Neslihan Gursoy R., Benita S. (2014). Self-emulsifying drug delivery systems (SEDDS) for improved oral delivery of lipophilic drugs. Biomed. Pharmacother..

[61] Alshehri S., Imam S.S., Hussain A., Altamimi M.A., Alruwaili N.K., Alotaibi F., Alanazi A., Shakeel F. (2020). Potential of solid dispersions to enhance solubility, bioavailability, and therapeutic efficacy of poorly water-soluble drugs: Newer formulation techniques, current marketed scenario and patents. Drug Deliv..

[62] Rogers R.D., Seddon K.R. (2003). Ionic liquids—Solvents of the future?. Science.

[63] Zhou S., Huang G., Chen G. (2019). Synthesis and biological activities of local anesthetics. RSC Adv..

[64] Hough W.L., Smiglak M., Rodríguez H., Swatloski R.P., Spear S.K., Daly D.T., Pernak J., Grisel J.E., Carliss R.D., Soutullo M.D. (2007). The third evolution of ionic liquids: Active pharmaceutical ingredients. New J. Chem..

[65] Araújo J.M., Florindo C., Pereiro A.B., Vieira N.S., Matias A.A., Duarte C.M., Rebelo L.P.N., Marrucho I.M. (2014). Cholinium-based ionic liquids with pharmaceutically active anions. RSC Adv..

[66] Lee P.Y., Wong K.K. (2011). Nanomedicine: A new frontier in cancer therapeutics. Curr. Drug Deliv..

[67] McCord J., Lang J.R., Hill D., Strynar M., Chernoff N. (2018). pH dependent octanol–water partitioning coefficients of microcystin congeners. J. Water Health.

[68] Sahbaz Y., Williams H.D., Nguyen T.-H., Saunders J., Ford L., Charman S.A., Scammells P.J., Porter C.J.H. (2015). Transformation of poorly water-soluble drugs into lipophilic ionic liquids enhances oral drug exposure from lipid-based formulations. Mol. Pharm..

[69] Florindo C., de Araújo J.M.M., Alves F., Matos C., Ferraz R., Prudêncio C., Noronha J.P., Petrovski Ž., Branco L., Rebelo L.P.N. (2013). Evaluation of solubility and partition properties of ampicillin-based ionic liquids. Int. J. Pharm..

[70] Egorova K.S., Gordeev E.G., Ananikov V.P. (2017). Biological Activity of Ionic Liquids and Their Application in Pharmaceutics and Medicine. Chem. Rev..

[71] Dharamdasani V., Mandal A., Qi Q.M., Suzuki I., Bentley M.V.L.B., Mitragotri S. (2020). Topical delivery of siRNA into skin using ionic liquids. J. Control. Release.

[72] Gruss M. (2021). Aspects for Developing and Processing Solid Forms. Solid State Development and Processing of Pharmaceutical Molecules: Salts Cocrystals, and Polymorphism.

[73] Chistyakov D., Sergeev G. (2020). The Polymorphism of Drugs: New Approaches to the Synthesis of Nanostructured Polymorphs. Pharmaceutics.

[74] Datta S., Grant D.J. (2004). Crystal structures of drugs: Advances in determination, prediction and engineering. Nat. Rev. Drug Discov..

[75] Kara D.D., Rathnanand M. (2022). Cocrystals and Drug–Drug Cocrystals of Anticancer Drugs: A Perception towards Screening Techniques, Preparation, and Enhancement of Drug Properties. Crystals.

[76] Waskewitz P. (2007). Machine vision in manufacturing. Handbook of Machine Vision.

[77] Pedro S.N., Freire C.S., Silvestre A.J., Freire M.G. (2021). Deep Eutectic Solvents and Pharmaceuticals. Encyclopedia.

[78] Torimoto T., Tsuda T., Okazaki K.I., Kuwabata S. (2010). New frontiers in materials science opened by ionic liquids. Adv. Mater..

[79] Prodius D., Mudring A.-V. (2018). Rare earth metal-containing ionic liquids. Coord. Chem. Rev..

[80] Kleitz F. (2009). Ordered Microporous and Mesoporous Materials. Nanoscale Materials in Chemistry.

[81] Benjamin M., Manoj D., Thenmozhi K., Bhagat P.R., Saravanakumar D., Senthilkumar S. (2017). A bioinspired ionic liquid tagged cobalt-salophen complex for nonenzymatic detection of glucose. Biosens. Bioelectron..

[82] Gorle D.B., Ponnada S., Kiai M.S., Nair K.K., Nowduri A., Swart H.C., Ang E.H., Nanda K.K. (2021). Review on recent progress in metal–organic framework-based materials for fabricating electrochemical glucose sensors. J. Mater. Chem. B.

[83] López M.S.P., Mecerreyes D., López-Cabarcos E., López-Ruiz B. (2006). Amperometric glucose biosensor based on polymerized ionic liquid microparticles. Biosens. Bioelectron..

[84] Peng D., Picchioni F. (2020). Prediction of toxicity of Ionic Liquids based on GC-COSMO method. J. Hazard. Mater..

[85] Almutairi S.M., El-Sayed W.S., Sahu P.K., Thasneema K.K. (2021). Some 4-dimethylaminopyridinium-based ionic liquids and/or salts. Part I: Efficient green ultrasound synthesis, characterization, in silico prediction analysis, toxicity and antimicrobial evaluation. Arab. J. Chem. Environ. Res..

[86] Kurnia K.A., Sintra T., Neves C.S., Shimizu K., Canongia Lopes J.N., Gonçalves F.J.M., Ventura S., Freire M., Santos L., Coutinho J.A.P. (2014). The effect of the cation alkyl chain branching on mutual solubilities with water and toxicities. Phys. Chem. Chem. Phys..

[87] Gonçalves A., Paredes X., Cristino A., Santos F., Queirós C. (2021). Ionic Liquids—A Review of Their Toxicity to Living Organisms. Int. J. Mol. Sci..

[88] Wang B., Song A., Feng L., Ruan H., Li H., Dong S., Hao J. (2015). Tunable amphiphilicity and multifunctional applications of ionic-liquid-modified carbon quantum dots. ACS Appl. Mater. Interfaces.

[89] Li D.Y., Wang Y.Z., Zhao X.L., He X.W., Li W.Y., Zhang Y.K. (2014). Facile synthesis of ionic liquid functionalized silica-capped CdTe quantum dots for selective recognition and detection of hemoproteins. J. Mater. Chem. B.

[90] Nayl A.A., Arafa W.A.A., Ahmed I.M., Abd-Elhamid A.I., El-Fakharany E.M., Abdelgawad M.A., Gomha S.M., Ibrahim H.M., Aly A.A., Bräse S. (2022). Novel pyridinium based ionic liquid promoter for aqueous knoevenagel condensation: Green and efficient synthesis of new derivatives with their anticancer evaluation. Molecules.

[91] Wu H., Deng Z., Zhou B., Qi M., Hong M., Ren G. (2019). Improved transdermal permeability of ibuprofen by ionic liquid technology: Correlation between counterion structure and the physicochemical and biological properties. J. Mol. Liq..

[92] Clark K.D., Emaus M.N., Varona M., Bowers A.N., Anderson J.L. (2018). Ionic liquids: Solvents and sorbents in sample preparation. J. Sep. Sci..

[93] Greer A.J., Jacquemin J., Hardacre C. (2020). Industrial Applications of Ionic Liquids. Molecules.

[94] Shamshina J.L., Berton P., Wang H., Zhou X., Gurau G., Rogers R.D. (2018). Ionic liquids in pharmaceutical industry. Green Techniques for Organic Synthesis and Medicinal Chemistry.

[95] Stoimenovski J., MacFarlane D.R., Bica K., Rogers R.D. (2010). Crystalline vs. ionic liquid salt forms of active pharmaceutical ingredients: A position paper. Pharm. Res..

[96] Santos M.M., Raposo L.R., Carrera G.V.S.M., Costa A., Dionísio M., Baptista P.V., Fernandes A.R., Branco L.C. (2019). Ionic Liquids and Salts from Ibuprofen as Promising Innovative Formulations of an Old Drug. ChemMedChem.

[97] Magina S., Barros-Timmons A., Ventura S.P., Evtuguin D.V. (2021). Evaluating the hazardous impact of ionic liquids–challenges and opportunities. J. Hazard. Mater..

[98] Curreri A.M., Mitragotri S., Tanner E.E. (2021). Recent advances in ionic liquids in biomedicine. Adv. Sci..

